# Hepatocyte growth factor regulates HLX1 gene expression to modulate HTR-8/SVneo trophoblast cells

**DOI:** 10.1186/1477-7827-10-83

**Published:** 2012-09-18

**Authors:** Hai-Ying Liu, Xue-Qin Jia, Ling-Xue Gao, Yu-Yan Ma

**Affiliations:** 1 Department of Obstetrics and Gynecology, Qilu Hospital of Shandong University, Jinan, China; 2 Department of Obstetrics and Gynecology, Rizhao People’s Hospital, Rizhao, China; 3 Department of Obstetrics and Gynecology, Maternal and Child Healthcare Hospital of Qingdao, Qingdao, China

**Keywords:** Hepatocyte growth factor, Trophoblast, Cell invasion, RNA interference

## Abstract

**Background:**

Paracrine signaling of the hepatocyte growth factor (HGF) cytokine plays an important role in survival and invasion ability of placental trophoblasts. However, the intracellular factors and biological pathways underlying these responses remain unclear.

**Methods:**

This study investigated whether HGF affected the expression of homeobox gene *HLX1*, which is principally expressed in reproductive tissues and in some immune cells, and evaluated the implications of such in the HGF-induced human trophoblast cell line HTR-8/SVneo.

**Results:**

HGF was found to up-regulate both HLX1 mRNA and protein levels. Transient transfection of small interfering RNA (siRNA) targeting *HLX1* abrogated its induction by HGF. Functionally, *HLX1* siRNA not only reduced the growth and invasion capacities of HTR-8/SVneo cells at the basal level, but also inhibited these responses induced by HGF treatment.

**Conclusions:**

HLX1 is an essential downstream signaling component of HGF that leads to growth and invasiveness of trophoblast cells.

## Background

Trophoblasts are specialized placental cells that facilitate embryo implantation and subsequently differentiate to form the placenta. Both processes depend extensively on complex and dynamic molecular crosstalk between fetal and maternal tissues. Upon blastocyst implantation, trophoblasts begin to differentiate into two distinct subpopulations, namely the villous trophoblasts (VTs) and extravillous trophoblasts (EVTs). The VTs function mainly to exchange gas and nutrients between the mother and the fetus, while the EVTs invade into the maternal uterus and establish feto-placental vasculature [1]. Mounting evidence has implicated various cytokines and growth factors in regulating the growth and invasion ability of EVTs; these include hepatocyte growth factor (HGF), epidermal growth factor (EGF), transforming growth factor beta (TGFβ), insulin-like growth factor II (IGF-II) and interleukin β (IL-β) [1]. Among these factors, HGF is a particularly potent paracrine mitogen, motogen and morphogen, known to act on multiple cell types during various developmental and regenerative processes, including embryonic organogenesis, adult tissue regeneration after injury and wound healing [2].

HGF binding to the cell-surface receptor Met tyrosine kinase triggers a multi-component signal transduction pathway that leads to dynamic biological responses. Homozygous HGF gene knockout in transgenic mice resulted in severely impaired placental development and markedly reduced trophoblast numbers; ultimately, the embryos produced in HGF^−/−^ mothers suffered prenatal death [3,4]. Intriguingly, a similar placental failure was observed in mice containing homozygous mutants of the c-met gene, which supported the hypothesized role of HGF/c-met signaling in physiological placental development [5].

Human placenta is a rich source of HGF. The yield of HGF in mature placenta is approximately 100- to 200-fold higher than that obtained from human plasma, implying its significant role in placental functions [6]. During pregnancy, placental HGF expression is restricted to the villous core mesenchymal cells, and c-met is mainly expressed on the surfaces of trophoblasts and endothelial cells [7,8]. These distinctive expression patterns imply that HGF may serve as a paracrine mediator of trophoblasts and endothelial cells to control placental development and growth. In agreement with this idea, low maternal HGF levels in humans have been correlated with pregnancies yielding infants small for their gestational age [9]. *In vitro* and *in vivo* molecular studies have revealed that HGF stimulates growth and invasion ability of trophoblasts [7,10-13], yet the underlying factors involved in the signaling mechanisms remain to be identified.

Homeobox gene transcription factors are a family of homeodomain-containing proteins that regulate cellular differentiation and organ development. HLX1 is a homeobox family member that is mainly expressed in VTs and EVTs of early-pregnancy placenta and in residual EVTs of full-term placenta [14]. HLX1 expression was found to be down-regulated in fetal growth-restricted (FGR) human placenta, as compared to that in normal growth placenta [15]. This finding suggested that unbalanced HLX1 expression may contribute to the pathogeneses of placental and trophoblastic diseases. The additional finding that the down-regulated HLX1 in FGR placenta was accompanied by reduced HGF expression [15] prompted us to examine the potential crosstalk between these two molecules. In this study, we used the EVT cell line HTR-8/SVneo to investigate HLX1 expression in response to HGF and small interfering RNA (siRNA) gene targeting to determine whether HGF-mediated HLX1 contributes to the biological processes induced by HGF.

## Methods

### Cell culture, HGF treatment and siRNA transfection

The human EVT cell line HTR-8/SVneo was generously provided by Dr. Charles H. Graham (Department of Anatomy & Cell Biology, Queen’s University at Kingston, Canada). Cells were cultured in RPMI-1640 medium (Hyclone, China) supplemented with 10% heat-inactivated fetal bovine serum (FBS; Sijiqing Biotec Co., China) in a sterile incubator at 37°C with 95% humidity and 5% CO_2_.

For HGF treatment, cells were grown to log phase, released from the culture dish by trypsinization (0.25% trypsin; Sigma, USA), and seeded into 6-well plates at 2 × 10^5^ cells/well. Twenty-four hours later, cells were starved in RPMI-1640 medium containing 0.5% FBS for 12 h and then treated with HGF (PeproTech, USA) at different concentrations for a further 48 h.

A DY-547-labeled control siRNA duplex (sense-strand sequence: 5'- UAGCGACUAAACACAUCAAUU-3') that targets no known human genes, and an siRNA pool containing four siRNA duplexes specifically targeting human *HLX1* sequences were purchased from Dharmacon (USA). The sense-strand sequences for the four HLX1 siRNA duplexes were as follows: HLX1-si1 5'-GAAAUUCAGUUCAGCAUCA-3'; HLX1-si2 5'-GGUUUGAGAUUCAGAAGUA-3'; HLX1-si3 5'-GAUCUCACUUCCCUGCUAA-3'; and HLX1-si4 5'-GGACGCGAGUGGUUCCGAA-3'. siRNA was transfected into HTR-8/SVneo cells using DharmaFECT1 reagent (Dharmacon) following the manufacturer’s instructions.

### Reverse transcription followed by quantitative real-;time (q)PCR

Total RNA was extracted from cells using Trizol reagent (Invitrogen, USA), according to the manufacturer’s protocol. Following quantification by UV spectrophotometer, 2 μg of total RNA was reverse transcribed into cDNA by using a commercially-available reverse transcription kit (Fermentas, Lithuania). The cDNA was then used as a template for subsequent qPCR analysis with SYBR^®^ Premix *Ex Taq*™ and the following primers (TaKaRa, Japan): β-actin (internal control, amplicon size of 188 bp): upstream 5'-TGGCACCCAGCACAATGAA-3′ and downstream 5′-CTAAGTCATAGTCCGCCTAGAAGCA-3′; *HLX1* (amplicon size of 175 bp): upstream 5′-CAGTTCAGCATCAGTTCCAAGACAC −3′ and downstream 5′-TCCGGCTTGGTCACGTACTTC-3′. Thermal cycling conditions for amplification were: one cycle of 95°C for 20 sec, followed by 45 cycles of 95°C for 5 sec, 60°C for 30 sec and 72°C for 10 sec. The relative gene expression was calculated using the 2^-ΔΔCt^ method, as previously described [16].

### Western immunoblot

Cells were lysed in sodium dodecyl sulfate (SDS) lysis buffer (Beyotime, China) and the total protein concentration was determined using the BCA protein assay kit (Beyotime), following the manufacturer’s instructions. An 80 μg aliquot of total protein from each sample was separated by 10% SDS-polyacrylamide gel electrophoresis (PAGE) and electrotransferred to a nitrocellulose membrane. After blocking with 5% skim milk at room temperature for 3 h, the membrane was incubated with one of the following primary antibodies at 4°C overnight: rabbit anti-HLX1 (0.25 μg/μL; Abcam, USA) or mouse anti-β-actin (0.1 μg/μL; Santa Cruz Biotech., USA). After washing, the membrane was incubated with horseradish peroxidase-conjugated anti-rabbit secondary antibody (1:1000; Zymed, USA) at room temperature for 90 min and the signal was developed with enhanced chemiluminescent substrate and detected on a Kodak gel imaging station. Immunoreactive signals were quantified using JEDA imaging and analysis software (version 3.3; JEDA Science-Tech., China), and presented as the intensity ratio of HLX1 signal to β-actin signal.

### Flow cytometry

At 24 h after transfection of HTR-8/SVneo cells with different concentrations of DY-547-tagged control siRNA, cells were trypsinized, resuspended in ice-cold sterile phosphate buffered saline (PBS), and analyzed by flow cytometry. The percentage of DY-547-positive cells was used as an indicator of transfection efficiency.

### Cell viability by MTT assay

HTR-8/SVneo cells at log phase were seeded into 96-well plates at 5 × 10^3^ cells/well. After overnight incubation, cells were transfected with either control or *HLX1* siRNA (time 0; five replicates for each condition). At 0, 24, 48, 72 and 96 h post-transfection, the cell viability was determined using MTT reagent (Gibco, USA) and reading absorbance (A) at 490 nm. The growth inhibition was calculated as [(1-A_Experiment_/A_Blank well_) × 100%].

### Gelatin zymography of matrix metalloproteinases (MMP)2 and MMP9

Confluent cultures of trophoblast cells were starved for 24 h in RPMI-1640 medium supplemented with 0.5% FBS. Following starvation, the cells were stimulated with 20 ng/mL HGF in serum-free media for a further 24 h. The mock-stimulated control cells were treated with only serum-free medium, without HGF added. For experiments involving HLX1 down-regulation with HGF stimulation, cells were transfected with siRNA prior to serum starvation and HGF stimulation. Following the treatment, conditioned medium was harvested from the cells and a 10 μL aliquot was mixed with an equal volume of 2× Laemmli sample buffer and separated on an 8% SDS-PAGE gel containing 0.1% gelatin. After electrophoresis, gels were soaked and washed twice in 2.5% Triton X-100 for 45 min each at 37°C and then incubated in a buffer containing 50 mM Tris buffer, pH 7.5, 200 nM NaCl, 5 mM CaCl_2_ 1 μM ZnCl_2_, and 0.2% Brij 35 at 37°C overnight. After 12 h, the gels were stained for 4 h with 0.25% Coomassie Brilliant Blue G-250 in a solution of 10% acetic acid and 45% methanol, and then destained for 30 minutes in the same solution without Coomassie. The Coomassie-stained gel was imaged using the Kodak gel imaging station and analyzed by JEDA imaging and analysis software. Conditioned medium from human fibrosarcoma cells HT1080 was collected for use as the positive control for MMP2 and MMP9 activity.

### Transwell invasion assay

The high concentration Matrigel matrix (BD Biosciences, USA) was thawed at 4°C, diluted 1:3 in RPMI-1640 medium, coated on the upperside of a transwell insert (8 μm pore size; Corning, USA) at 100 μL/insert and incubated at 37°C for 30 min. Meanwhile, cells were resuspended in serum-free RPMI-1640 medium at 1 × 10^5^ cells/mL and 400 μL aliquots were deposited on the top of the transwell insert. To the bottom well, 1200 μL of RPMI-1640 medium with 20% FBS was added. The invasion assay was allowed to proceed at 37°C for 12 h. After the invasion period, the cells remaining on top were gently removed by a cotton swab, and those that had transversed to the bottom side of the insert were fixed in methanol and stained with hematoxylin. The insert membrane was then cut out and mounted onto glass slides with the bottom side facing upward. The invasive cells were imaged under a light microscope (IX81; Olympus, Japan) at 400 × magnification. Five random fields were imaged for each membrane and the cell numbers detected in each were averaged.

### Statistical analysis

Quantitative data are presented as mean ± standard deviation (SD) for at least three independent experiments. Differences between groups were evaluated by using one-way analysis of variance (ANOVA). All statistical analyses were carried out with SPSS software (version 11.0; SPSS Institute, USA). A *P*-value of <0.05 was considered statistically significant.

## Results

### HGF up-regulates the expression of HLX1 in HTR-8/SVneo cells

**Figure 1:**
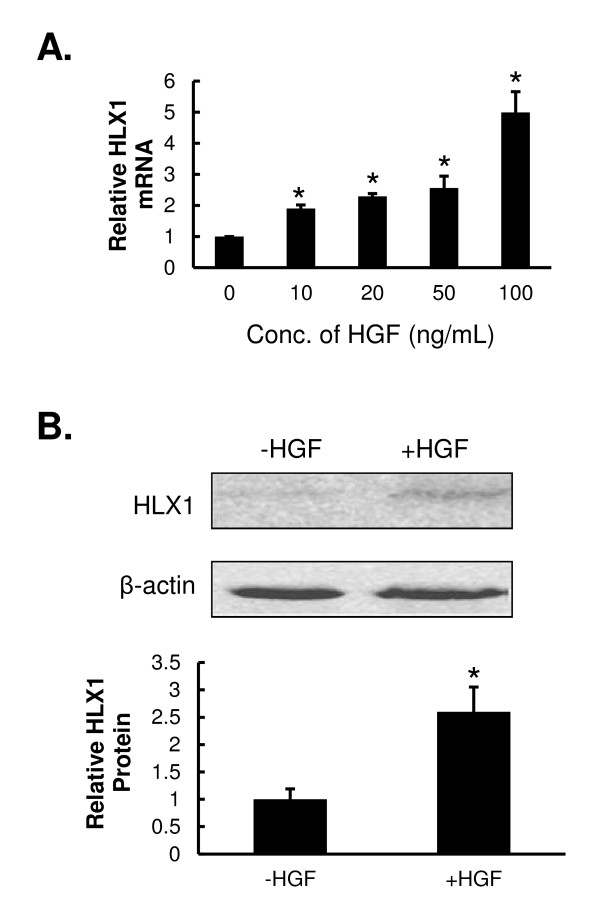
**HGF up-regulates the expression of HLX1 in HTR-8/SVneo cells.** (**A**) HTR-8/SVneo cells were treated with the indicated concentrations of HGF for 48 h. The steady-state mRNA level of HLX1 was examined by qPCR and normalized to a β-actin internal control. The ratio of HLX1/β-actin mRNA in cells treated with 0 ng/mL HGF was arbitrarily defined as 1. Data are presented as mean ± SD for three independent experiments. *P < 0.01, vs. 0 ng/mL HGF. (**B**) HTR-8/SVneo cells were treated with 0 ng/mL (−) or 20 ng/mL (+) HGF for 48 h. The protein level of HLX1 was determined by Western immunoblot and normalized to β-actin internal control. A representative gel image is presented in the upper panel and quantification of HLX1 to β-actin signal ratio from three independent experiments is shown in the lower panel. The ratio in –HGF cells was arbitrarily defined as 1. *P < 0.01, vs. 0 ng/mL HGF.

In response to increasing concentrations of HGF, the steady-state mRNA level of *HLX1* in HTR-8/SVneo cells showed a dose-dependent increase (Figure 1A), and significant difference was achieved with as little as 10 ng/mL HGF (*P* < 0.01). At 100 ng/mL HGF, the mRNA level of *HLX1* increased to almost five-fold more than that of the endogenous level in HTR-8/SVneo cells. For subsequent experiments, we chose the intermediate dose of HGF (20 ng/mL) that showed the most robust dose response among all HGF concentrations tested. Similar changes were also observed at the protein level of HLX1 (Figure 1B). When HTR-8/SVneo cells were treated with 20 ng/mL HGF, the protein level of HLX1 was approximately 2.6-fold higher than that in cells without HGF treatment (*P* < 0.01).

### Targeting HLX1 with siRNA abrogates basal expression and induction by HGF

**Figure 2:**
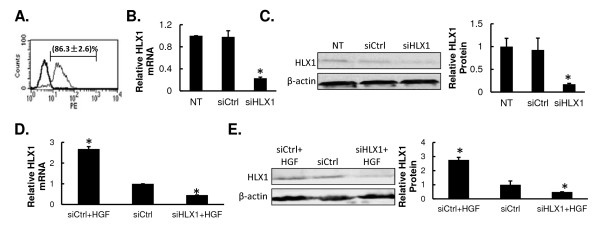
**Targeting HLX1 with siRNA abrogates its basal expression and induction by HGF.** (**A**) DY-547-labeled control siRNA (siCtrl) was transfected into HTR-8/SVneo cells for 24 h. The transfection efficiency, as determined by flow cytometry, is presented. Black line, non-transfected (NT) cells; gray line, siCtrl cells. (**B**) The steady-state mRNA level of HLX1 in indicated HTR-8/SVneo cells was examined by qPCR and calculated as the ratio of HLX1 to ß-actin. (**C**) The protein level of HLX1 in indicated HTR-8/SVneo cells was examined by Western immunoblot. A representative gel image is presented on the left and quantifications of HLX1 to Î²-actin signal ratio on the right.(**D**) HTR-8/SVneo cells were transfected with either siCtrl or siHLX1 and treated with or without 20 ng/mL HGF. The steady-state mRNA level of HLX1 was examined by qPCR and calculated as the ratio of HLX1 to Β-actin. (**E**) HTR-8/SVneo cells were treated as in (**D**) and the protein level of HLX1 was examined by Western immunoblot. A representative gel image is presented on the left and quantifications of HLX1 to Î²-actin signal ratio on the right. Quantitative data are presented as mean Â± SD for three independent experiments. For (**B**) and (**C**), the relative ratio in NT cells arbitrarily defined as 1. *P < 0.01, vs. NT and siCtrl cells. For (**D**) and (**E**), the relative ratio in siCtrl-transfected cells arbitrarily defined as 1. *P < 0.01, vs. siCtrl cells.

Given the apparent HGF-inducible nature of HLX1, we investigated the significance of HLX1 in HGF-induced biological processes. A loss-of-function approach was applied by knocking down the expression of HLX1 with siRNA-mediated gene targeting. We first examined the efficiency of siRNA transfection by using the fluorescent probe DY-547-tagged non-targeting control siRNA (siCtrl). By transfecting 10, 50 and 100 nM siRNA, we found that the 100 nM concentration produced the highest transfection efficiency (86.3 ± 2.6%; Figure 2A). Therefore, this dosage was used for all subsequent experiments.

At 48 h after siRNA transfection, we examined the mRNA and protein levels of HLX1 by qPCR and Western blot, respectively. As shown in Figure 2B and 2C, transfection with the HLX1 siRNA pool (siHLX1) significantly reduced HLX1 expression on both mRNA and protein levels, as compared to the levels detected in siCtrl-transfected or non-transfected (NT) HTR-8/SVneo cells (*P* < 0.01). In addition to down-regulating endogenous HLX1, siHLX1 also inhibited its HGF-induced expression levels in both mRNA and protein (Figure 2D and 2E). HGF treatment of siCtrl cells led to an approximate 2.7-fold increase in HLX1 mRNA and 2.8-fold increase in HLX1 protein. In contrast, siHLX1 transfected cells presented reduced HLX1 mRNA (by 55%) and protein (by 52%), even in the presence of HGF (*P* < 0.01) suggesting that HLX1 siRNA has a more dominant effect over HGF in regulating HLX1 expression. These three treatments provide us a model where the intracellular HLX1 levels vary from each other, therefore allowing us to further observe the significance of HLX1 in cellular behaviors as detailed below.

### Targeting HLX1 inhibits basal and HGF-induced cell growth

**Figure 3:**
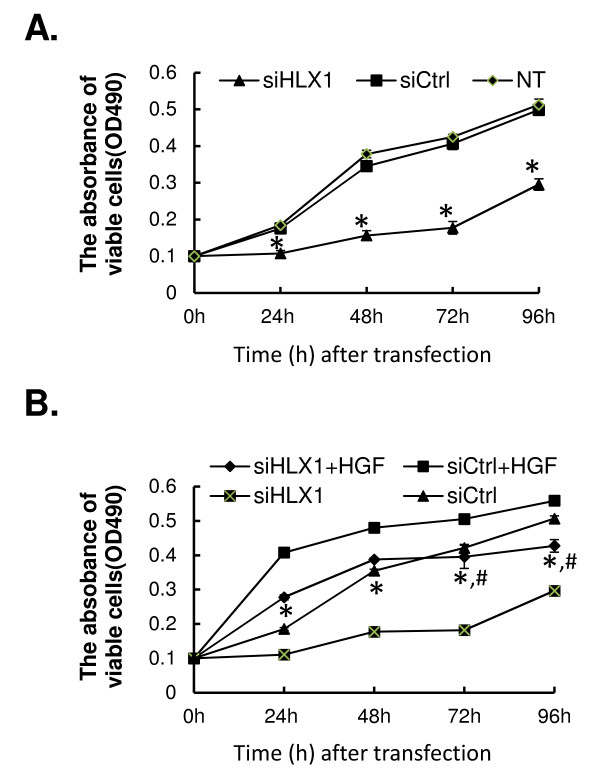
**Targeted silencing of HLX1 inhibits basal and HGF-induced cell growth. **(**A**) HTR-8/SVneo cells were either not transfected (NT), or transfected with siCtrl or siHLX and cell growth at 0, 24, 48, 72 and 94 h after transfection was determined by MTT assay. (**B**) HTR-8/SVneo cells were transfected with either siCtrl or siHLX1 at time 0, and treated with or without 20 ng/mL HGF. Cell growth at 0, 24, 48, 72 and 94 h after transfection was determined by MTT assay with the absorbance of viable cells read at 490 nm (OD490). All quantitative data are presented as mean ± SD for three independent experiments. *P < 0.01, as compared to NT cells or siCtrl-transfected cells (in **A**), and to siCtrl + HGF or siHLX1 cells (in **B**). ^#^*P* < 0.01, as compared to siHLX1 + HGF cells (in **B**).

HGF is an important mitogen and motogen for trophoblasts, essentially regulating their proliferation, apoptosis and migration/invasion capabilities [3,17,18]. To evaluate the significance of HLX1 in HGF-mediated biological processes, we first examined HGF-induced growth of cells transfected with either siCtrl or siHLX1 by using the MTT assay (Figure 3A). The same number of cells were transfected at time 0. Under basal conditions, namely without HGF treatment, both NT and siCtrl-transfected HTR-8/SVneo cells grew steadily within the 96 h post-transfection observation period. However, this growth was dramatically reduced in the siHLX1-transfected cells, and a significant difference was found to have occurred as early as 24 h after siRNA transfection. The largest difference was observed at 72 h after transfection, with growth inhibition of 58.1 ± 4.4% (*P* < 0.01).

In response to HGF treatment, siCtrl cells grew significantly slower than siCtrl + HGF cells, but faster than siHLX1 cells, over the 96 h observation period following siRNA transfection (*P* < 0.01; Figure 3B). The growth observed for siHLX1 + HGF cells, however, was not dramatically different from siCtrl cells at 24 and 48 h post-transfection, but was significantly reduced at 72 and 96 h post-transfection (*P* < 0.01).

### Targeting HLX1 reduces HGF-induced cell invasiveness and production of MMP2

**Figure 4:**
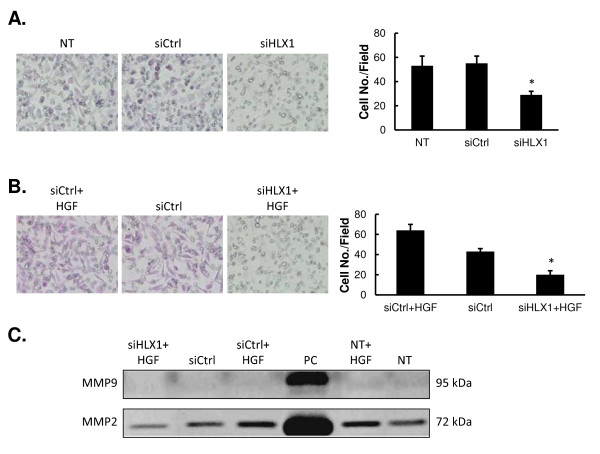
**Targeting HLX1 reduces the cell invasion and the production of MMP2 in response to HGF.** (**A**) HTR-8/SVneo cells were either not transfected (NT), or transfected with siCtrl or siHLX. The invasion of transfected cells across matrigel was determined by a transwell assay. Images of representative invasive cells are shown on the left, and quantifications are shown on the right. (**B**) HTR-8/SVneo cells were transfected with either siCtrl or siHLX1 and treated with or without 20 ng/mL HGF. The invasion of transfected cells across matrigel was determined by a transwell assay. Images of representative invasive cells are shown on the left, and quantifications are shown on the right. All quantitative data are presented as mean ± SD for three independent experiments. *P < 0.01, as compared to the other two groups. (**C**) HTR-8/SVneo cells were treated as indicated. The MMP2 and MMP9 levels in the conditioned medium from the cells were measured by gelatin zymography; conditioned medium collected from HT1080 cells were used as a positive control (PC).

Next, the effects of knocking down HLX1 were determined by the invasive properties of trophoblasts. By imaging and quantifying the cells invading through the matrigel, we found that siHLX1 transfection not only reduced the basal invasiveness of HTR-8/SVneo cells (*vs.* NT or siCtrl-transfected cells, *P* < 0.01; Figure 4A), but also significantly inhibited the HGF-induced invasion properties (*vs.* compared to siCtrl-transfected cells or siCtrl-transfected cells treated with HGF, *P* < 0.01; Figure 4B).

To understand the regulatory mechanism underlying HLX1 associated HGF-induced cell invasiveness, we focused on two matrix metalloproteinase molecules, MMP2 and MMP9, that are characterized as downstream targets for HGF and important regulators of cell migration/invasion [19]. By gelatin zymography analysis, we found that the MMP2 activity in the conditioned medium was significantly enhanced upon HGF treatment, but not affected by siCtrl transfection (siCtrl *vs*. siCtrl + HGF or NT *vs*. NT + HGF samples, *P* < 0.01; siCtrl *vs*. NT or siCtrl + HGF *vs*. NT + HGF samples, *P* > 0.05; Figure 4C). Transfection of siHLX1, however, significantly reduced the MMP2 activity in the conditioned medium (*vs*. all other samples, *P* < 0.01). The MMP9 activity was very low in the conditioned medium, as compared with that in the conditioned medium from HT1080 cells, and no significant variations were found between HTR-8/SVneo cells treated with HGF or transfected with siHLX1.

## Discussion

In this study, we identified HLX1 as a downstream target for HGF signaling in the extravillous trophoblast cell line HTR-8/SVneo. Our analysis provided experimental evidence that HLX1 is essential for the *in vitro* growth and invasion properties of HTR-8/SVneo cells under regular culture conditions or in response to HGF treatment.

HTR-8/SVneo cells were originally derived from first-trimester villous explants and immortalized by transfection with the pSV3neo vector containing the early region of SV40 encoding the simian virus 40 large T antigen [20]. Despite being immortalized, these cells retain remarkably similar gene and protein expression profiles as the parental mortal HTR-8 cells. The characteristic cell-type molecules are cytokeratins 18 and 8, human placental lactogen (hPL), human chorionic gonadotropin (hCG), human leukocyte antigen G (HLA-G), and type IV collagenase. Furthermore, like the source HTR-8 cells, HTR-8/SVneo cells lack the capability to grow in soft agar and produce no tumors following subcutaneous injection into nude mice [20,21]. These features make HTR-8/SVneo cells an ideal *in vitro* model for studying the biology of normal trophoblasts.

The HTR-8/SVneo cells in our study responded to HGF treatment with increased cell growth and enhanced invasion across matrigel. Both of these features are consistent with the biological functions known for HGF [6,18,22]. The invasion capacity of EVTs has been the focus of extensive studies to date since decreased trophoblast invasion is a characteristic feature of preeclampsia, a dangerous complication of pregnancy that results in significant maternal and fetal morbidity and mortality. Lala *et al.* reported that HGF enhances the *in vitro* invasiveness of trophoblasts by approximately 4-fold [23]. Likewise, Dokras *et al*. demonstrated that expression of recombinant (r)HGF significantly increased the invasive activity of cytotrophoblasts isolated from all three trimesters [22]. Finally, Kauma *et al.* observed an HGF dose-dependent increase in the invasiveness of ED27 trophoblast cells across type I collagen [18].

Concomitant with the increased growth and invasion in response to HGF, we also observed a dose-dependent up-regulation of HLX1, suggesting that HLX1 is a downstream target of HGF. In hematopoietic progenitor cells, HLX1 is an important regulator of cell proliferation, differentiation and migration in response to growth factors or cytokines [24,25]. The common process of cell growth and migration/invasion that are regulated by both HGF and HLX1 prompted us to test whether HLX1 was able to mediate the growth and invasion responses to HGF in trophoblasts. By using siRNA-mediated gene targeting, we successfully knocked down endogenous HLX1, and found that HGF-induced HLX1 was also abrogated. Following siRNA transfection, we found that knocking down HLX1 not only significantly reduced cell growth but also inhibited the invasiveness of HTR-8/SVneo cells in response to HGF, suggesting that HLX1 is an essential mediator for HGF-induced trophoblast growth and invasion. In addition, these reductions also occurred in the absence of HGF treatment, implying that the endogenous level of HLX1, although it can be further increased by HGF, is necessary to maintain the basal growth and invasiveness of HTR-8/SVneo cells.

To gain an even further understanding of the mechanisms underlying HLX1-regulated cell invasion, we examined MMP2 and MMP9. The matrix metalloproteinases are critical mediators of extracellular matrix (ECM) degradation. Several studies have identified the significant involvement of both MMP2 and MMP9 in trophoblast invasion of the uterus [26,27]. In our study, we found that HGF stimulated the production of MMP2 and that this response was dramatically reduced upon knocking down HLX1. The activity of MMP9, on the other hand, was not significantly altered by either HGF treatment or HLX1 knock-down. This result indicated that MMP2, but not MMP9, is likely a particular MMP that contributes to the HGF- and HLX1-induced trophoblast invasion property. It is worthwhile to further study whether HLX1 directly acts on the MMP2 promoter or function indirectly through some other transcription factor.

Recently, Rajaraman *et al.* published their work on the significance of HLX1 in mediating HGF-induced migration of human trophoblasts [28]. Using two different human EVT cell lines, SGHPL-4 and HTR-8/SVneo, this group showed that HLX1 is induced by HGF, consistent with our findings. Interestingly, only the migration assay, but not the invasion assay, showed functional significance of HLX1. At this point, it is unclear what has caused the discordant observations in HLX1-mediated invasion between their study and ours. However, some important differences in study design exist: 1) Rajaraman *et al.* did not investigate the functional significance of endogenous HLX1, without HGF treatment, in cell invasion; and 2) the HGF dose used was different, albeit not to a great extent (Rajaraman *et al.*: 10 ng/mL and our study: 20 ng/mL). Considering the differential results obtained from the two studies, it is important to extend future analysis to more trophoblast cell lines, and even animal models.

## Conclusions

In summary, we have shown that HLX1 is a downstream mediator of HGF-induced growth and invasion properties of the HTR-8/SVneo trophoblast cells. Given that both HGF and HLX1 are significantly associated with normal placental development and pathogenesis of placenta-related diseases, understanding the crosstalk between these two molecules may help to develop therapeutic approaches that will benefit normal pregnancy and treat pathological situations.

## Competing interests

The authors declare that they have no competing interests.

## Authors’ contributions

H-YL conceived of and designed the study, carried out the cell culture, HGF treatment and siRNA transfection experiments, and drafted the manuscript. L-XG carried out the reverse transcription followed by quantitative real-time PCR and Western immunoblot. X-QJ carried out the cell viability by MTT assay, transwell invasion assay and performed the statistical analysis. Y-YM participated in the experimental design and coordination, and helped to draft the manuscript. All authors read and approved the final manuscript.

## References

[B1] Lunghi L Ferretti ME Medici S Biondi C Vesce F Control of human trophoblast function Reprod Biol Endocrinol 2007 5 6 10.1186/1477-7827-5-6 17288592 PMC1800852

[B2] Nakamura T Sakai K Nakamura T Matsumoto K Hepatocyte growth factor twenty years on: Much more than a growth factor J Gastroenterol Hepatol 2011 26 Suppl 1 188 202 21199531 10.1111/j.1440-1746.2010.06549.x

[B3] Uehara Y Minowa O Mori C Shiota K Kuno J Noda T Kitamura N Placental defect and embryonic lethality in mice lacking hepatocyte growth factor/scatter factor Nature 1995 373 6516 702 705 10.1038/373702a0 7854453

[B4] Schmidt C Bladt F Goedecke S Brinkmann V Zschiesche W Sharpe M Gherardi E Birchmeier C Scatter factor/hepatocyte growth factor is essential for liver development Nature 1995 373 6516 699 702 10.1038/373699a0 7854452

[B5] Bladt F Riethmacher D Isenmann S Aguzzi A Birchmeier C Essential role for the c-met receptor in the migration of myogenic precursor cells into the limb bud Nature 1995 376 6543 768 771 10.1038/376768a0 7651534

[B6] Wolf HK Zarnegar R Oliver L Michalopoulos GK Hepatocyte growth factor in human placenta and trophoblastic disease Am J Pathol 1991 138 4 1035 1043 1849357 PMC1886112

[B7] Saito S Sakakura S Enomoto M Ichijo M Matsumoto K Nakamura T Hepatocyte growth factor promotes the growth of cytotrophoblasts by the paracrine mechanism J Biochem 1995 117 3 671 676 7629039 10.1093/oxfordjournals.jbchem.a124761

[B8] Clark DE Smith SK Sharkey AM Sowter HM Charnock-Jones DS Hepatocyte growth factor/scatter factor and its receptor c-met: localisation and expression in the human placenta throughout pregnancy J Endocrinol 1996 151 3 459 467 10.1677/joe.0.1510459 8994391

[B9] Aoki S Hata T Manabe A Miyazaki K Decreased maternal circulating hepatocyte growth factor (HGF) concentrations in pregnancies with small for gestational age infants Hum Reprod 1998 13 10 2950 2953 10.1093/humrep/13.10.2950 9804260

[B10] Cartwright JE Holden DP Whitley GS Hepatocyte growth factor regulates human trophoblast motility and invasion: a role for nitric oxide Br J Pharmacol 1999 128 1 181 189 10.1038/sj.bjp.0702757 10498850 PMC1571601

[B11] Somerset DA Li XF Afford S Strain AJ Ahmed A Sangha RK Whittle MJ Kilby MD Ontogeny of hepatocyte growth factor (HGF) and its receptor (c-met) in human placenta: reduced HGF expression in intrauterine growth restriction Am J Pathol 1998 153 4 1139 1147 10.1016/S0002-9440(10)65658-1 9777945 PMC1853066

[B12] Patel Y Kim H Rappolee DA A role for hepatocyte growth factor during early postimplantation growth of the placental lineage in mice Biol Reprod 2000 62 4 904 912 10.1095/biolreprod62.4.904 10727259

[B13] Nasu K Sugano T Matsui N Narahara H Kawano Y Miyakawa I Expression of hepatocyte growth factor in cultured human endometrial stromal cells is induced through a protein kinase C-dependent pathway Biol Reprod 1999 60 5 1183 1187 10.1095/biolreprod60.5.1183 10208981

[B14] Rajaraman G Murthi P Quinn L Brennecke SP Kalionis B Homeodomain protein HLX is expressed primarily in cytotrophoblast cell types in the early pregnancy human placenta Reprod Fertil Dev 2008 20 3 357 367 10.1071/RD07159 18402755

[B15] Murthi P Doherty V Said J Donath S Brennecke SP Kalionis B Homeobox gene HLX1 expression is decreased in idiopathic human fetal growth restriction Am J Pathol 2006 168 2 511 518 10.2353/ajpath.2006.050637 16436665 PMC1606485

[B16] Livak KJ Schmittgen TD Analysis of relative gene expression data using real-time quantitative PCR and the 2(−Delta Delta C(T)) Method Methods 2001 25 4 402 408 10.1006/meth.2001.1262 11846609

[B17] Dash PR Whitley GS Ayling LJ Johnstone AP Cartwright JE Trophoblast apoptosis is inhibited by hepatocyte growth factor through the Akt and beta-catenin mediated up-regulation of inducible nitric oxide synthase Cell Signal 2005 17 5 571 580 10.1016/j.cellsig.2004.09.015 15683732

[B18] Kauma SW Bae-Jump V Walsh SW Hepatocyte growth factor stimulates trophoblast invasion: a potential mechanism for abnormal placentation in preeclampsia J Clin Endocrinol Metab 1999 84 11 4092 4096 10.1210/jc.84.11.4092 10566655

[B19] Shin JC Moon HB Lee JH Yang DE Lee G Lee Y Lee JS Kim CY Kim SP Influence of Hepatocyte Growth Factor on Matrix Metalloproteinase Expression in HT cell line Korean J Obstet Gynecol 2001 44 12 2257 2262

[B20] Graham CH Hawley TS Hawley RG MacDougall JR Kerbel RS Khoo N Lala PK Establishment and characterization of first trimester human trophoblast cells with extended lifespan Exp Cell Res 1993 206 2 204 211 10.1006/excr.1993.1139 7684692

[B21] Shiverick KT King A Frank H Whitley GS Cartwright JE Schneider H Cell culture models of human trophoblast II: trophoblast cell lines--a workshop report Placenta 2001 22 Suppl A S104 S106 11312640 10.1053/plac.2001.0647

[B22] Dokras A Gardner LM Seftor EA Hendrix MJ Regulation of human cytotrophoblast morphogenesis by hepatocyte growth factor/scatter factor Biol Reprod 2001 65 4 1278 1288 10.1095/biolreprod65.4.1278 11566754

[B23] Lala PK Chakraborty C Factors regulating trophoblast migration and invasiveness: possible derangements contributing to pre-eclampsia and fetal injury Placenta 2003 24 6 575 587 10.1016/S0143-4004(03)00063-8 12828917

[B24] Kehrl JH Deguchi Y Potential roles for two human homeodomain containing proteins in the proliferation and differentiation of human hematopoietic progenitors Leuk Lymphoma 1993 10 3 173 176 10.3109/10428199309145879 8106064

[B25] Rajaraman G Murthi P Leo B Brennecke SP Kalionis B Homeobox gene HLX1 is a regulator of colony stimulating factor-1 dependent trophoblast cell proliferation Placenta 2007 28 10 991 998 10.1016/j.placenta.2007.03.011 17532041

[B26] Castellucci M De Matteis R Meisser A Cancello R Monsurro V Islami D Sarzani R Marzioni D Cinti S Bischof P Leptin modulates extracellular matrix molecules and metalloproteinases: possible implications for trophoblast invasion Mol Hum Reprod 2000 6 10 951 958 10.1093/molehr/6.10.951 11006325

[B27] Jovanovic M Stefanoska I Radojcic L Vicovac L Interleukin-8 (CXCL8) stimulates trophoblast cell migration and invasion by increasing levels of matrix metalloproteinase (MMP)2 and MMP9 and integrins alpha5 and beta1 Reproduction 2010 139 4 789 798 10.1530/REP-09-0341 20133364

[B28] Rajaraman G Murthi P Brennecke SP Kalionis B Homeobox gene HLX is a regulator of HGF/c-met-mediated migration of human trophoblast-derived cell lines Biol Reprod 2010 83 4 676 683 10.1095/biolreprod.109.078634 20554918

